# Laboratory Readiness and Response for SARS-Cov-2 in Indonesia

**DOI:** 10.3389/fpubh.2021.705031

**Published:** 2021-07-19

**Authors:** Dewi N. Aisyah, Chyntia A. Mayadewi, Gayatri Igusti, Logan Manikam, Wiku Adisasmito, Zisis Kozlakidis

**Affiliations:** ^1^Department of Epidemiology and Public Health, Institute of Epidemiology and Health Care, University College London, London, United Kingdom; ^2^Indonesia One Health University Network, Depok, Indonesia; ^3^Aceso Global Health Consultants Limited, London, United Kingdom; ^4^Faculty of Public Health, Universitas Indonesia, Depok, Indonesia; ^5^International Agency for Research on Cancer World Health Organization, Lyon, France

**Keywords:** Indonesia, pandemics, laboratory readiness, response, SARS-CoV-2

## Abstract

The laboratory diagnosis of SARS-CoV-2 infection comprises the informational cornerstone in the effort to contain the infections. Therefore, the ability to leverage laboratories' capacity in diagnostic testing and to increase the number of people being tested are critical. This paper reviews the readiness of Indonesian laboratories during the early months of the pandemic. It discusses the success of cross-sectoral collaboration among previously siloed national and sub-national government institutions, international development agencies, and private sector stakeholders. This collaboration managed to scale-up the COVID-19 referral laboratory network from one Ministry of Health NIHRD laboratory in the capital to 685 laboratories across 34 provinces. However, this rapid growth within 12 months since the first Indonesian case was discovered remained insufficient to cater for the constantly surging testing demands within the world's fourth most populous country. Reflecting on how other countries built their current pandemic preparedness from past emergencies, this paper highlights challenges and opportunities in workforce shortage, logistic distribution, and complex administration that need to be addressed.

## Introduction

Healthcare systems across the world have been placed under sustained pressure during the COVID-19 pandemic. The laboratory diagnosis of SARS-CoV-2 infection is the informational cornerstone in the efforts to contain the infections. Therefore, the needs to leverage the laboratories' capacity in diagnostic testing and to increase the number of people being tested quickly became critical issues globally (1–4). Several countries were able to conduct successful COVID-19 countermeasures through adequate laboratory diagnostic testing provision from the early stages of the pandemic. In Australia, its exemplary laboratory response was rooted in the previous infectious disease responses (for SARS-CoV-1 in 2003 and H1N1 Influenza pandemic in 2009), resulting to an integrated surveillance systems and automated testing platforms that were used and expanded, thus enabling laboratories to reach a larger testing capacity as needed (5). Similarly, Saudi Arabia's prior experiences (for H1N1 Influenza pandemic in 2009 and MERS in 2013) allowed for a greater preparedness and an effective response to the current pandemic (6). Additionally, South Korea is another example of a country that rapidly adapted its COVID-19 testing response, maintaining around 600 screening and testing centers through a close collaboration with the private sector, with its testing capacity reaching 110,000 tests as of November 2020 (7).

The utilization of rapid laboratory COVID-19 diagnosis can impact a number of activities, such as triage, the management of patients, as well as the prevention of sustained nosocomial infections (8). Additional laboratory tests, such as for neutrophilia and d-dimer levels, were found to be useful indicators for identifying hospitalized COVID-19 patients at a higher risk for illness severity and complications (9–11). From an epidemiological perspective, COVID-19 sequences allow the identification of variants of interest and variants of concern, such as fixed genomic mutations that might be associated with higher transmission and even mortality (12).

The number of COVID-19 confirmed cases continues to increase since its first reported cases in December 2019, reaching 115,578,778 total cumulative cases worldwide with 2,567,922 death by early March 2021 (13, 14). In Asia, the three countries reporting the highest numbers of cases are India (11,156,923), Iran (1,665,103), and Indonesia (1,361,098). Indonesia, the most populous country in Southeast Asia, reported the highest cumulative cases among others in the region (13, 14).

This paper focuses on Indonesia, the world's largest archipelagic country with highly fragmented geographical territory; world's fourth most populous nations with diverse culture and complex national-subnational decentralized governance that may create additional challenges in providing a harmonized laboratory diagnostic capacity. The focus in this manuscript is on the readiness of laboratories in Indonesia during the early months of the pandemic, as well as its resource requirements for subsequent surge in diagnostic capacity. We also describe the responses and policies related to COVID-19 testing in Indonesia, so that any relative weaknesses and strengths are reviewed within their respective context.

## Overview of the COVID-19 Situation in Indonesia

Indonesia announced its first laboratory confirmed SARS-CoV-2 case on March 2nd, 2020 which was in its capital, DKI Jakarta province (on Java island). Within the next 2 weeks, the disease had spread to adjacent provinces (West Java and Banten), and also outside of Java island, namely in the North Sumatera and East Kalimantan provinces. It only took 25 days to reach a further 19 provinces. By mid-April 2020, i.e., 45 days later, it had spread throughout all 34 provinces, while DKI Jakarta, West Java, Central Java, and South Sulawesi were the provinces reporting the highest cumulative cases nationally (15). The total laboratory confirmed cases by April 2020 (a month after the first case) reached 1,790, with 112 patients recovered, 170 deceased, and ~100 new cases per day, testifying to an active on-going transmission. A year later, by March 5th, 2021, the total confirmed cases reached 1,361,098, with 1,176,356 (86.4%) patients recovered, 36,897 (2.7%) patients deceased, and over 7,000 new cases per day. The number of active cases was 147,845, or 10.9% of the total cumulative cases (16).

## COVID-19 Diagnostic Laboratory Capacity in the Early Phase of the Pandemic

During the first month of the pandemic, the Ministry of Health (MoH) of the Republic of Indonesia appointed the National Institute of Health Research and Development (NIHRD) as the center of COVID-19 diagnostic laboratory activities. Thus, all specimens from all regions were brought to NIHRD, which is located in DKI Jakarta. Due to the high demands on the testing of specimens and the requirement for a faster analysis of the results, the MoH appointed a further 45 laboratories as national referral laboratories in mid-March 2020. This resulted into a sharp increase of cumulative tests: from under 1,000 in early March 2020, to more than 5,000 by that month's end, and reaching 35,000 by mid-April 2020. However, this surging laboratory diagnostic capacity was still insufficient to cater the need for community transmission surveillance of Indonesia's 270 million population.

During this initial phase, these 45 referral laboratories were only located in 22 out of 34 provinces. The remaining 12 provinces (Bengkulu, Lampung, East Nusa Tenggara, Central Kalimantan, East Kalimantan, North Kalimantan, Central Sulawesi, Southeast Sulawesi, Gorontalo, West Sulawesi, North Maluku, and West Papua) needed to send their samples through long hours of travel to laboratories in neighboring provinces. This added further pressure to the logistics capacity in ensuring the samples' safety and security (e.g., keeping samples in a good condition and appropriate temperature for prolonged periods) and led to inevitable reporting delays. Moreover, the available referral laboratories were facing several challenges, such as lack of appropriately trained laboratory staff; unavailability and inadequacy of materials, such as personal protective equipment; and speed and accuracy of RNA extraction (17) to meet the surging demand.

In terms of estimated daily testing capacity (by mid-April 2020), DKI Jakarta had the highest capacity of ~548 analyses per day, followed by East Java (301), Yogyakarta (238), Central Java (200), and West Java (148) (18). Since the largest testing capacity was available on Java Island, these laboratories had the heavy burden of conducting sample analyses from other islands too. While the testing capacity had increased, it was still insufficient, given the COVID-19's rapid transmission, which led to the eventual involvement of private laboratories and state veterinary laboratories to cater for an ever-increasing laboratory testing need (18).

One of the initial efforts by the government was to issue MoH regulations on diagnostic requirements and the list of referral laboratories in March 2020 (19) which clearly established diagnostic referral points. One month later, the MoH issued more regulations outlining the procedures and facility requirements that laboratories must meet to be able to analyze COVID-19 samples (20). This issuance allowed other laboratories that were not part of the initial COVID-19 laboratory reference list, to prepare, adjust their needs and operations accordingly and meet the prerequisites to join future iterations of the list. All of these actions facilitated the rapid surge in diagnostic capacity in later months (Supplementary Information). In addition, the National Government ensured the trust in laboratory diagnostic processes by establishing external quality assurance and conducting sample checks, both in national and subnational laboratories, to validate the appropriate use of molecular rapid test (TCM) and polymerase chain reaction (PCR) reagent tests (21).

On the other hand, the lack of laboratory staff that could be trained to carry out COVID-19 analysis was one of the major obstacles to these efforts. As a response the following were planned: (a) holding workshops to increase the capacity of laboratory technicians to collect swab specimens for PCR-based SARS-CoV-2 examinations; (b) conducting training for staff to assess the quality of the COVID-19 laboratories; (c) creating educational materials such as brochures and videos describing swab sampling and sending swab samples; and (d) providing technical guidance for the COVID-19 diagnostic laboratories. These efforts were supported by development partners operating in Indonesia, including the World Health Organization (WHO), United Nations Children's Fund (UNICEF), United States Agency for International Development (USAID), United States Centers for Disease Control and Prevention (CDC), Infectious Disease Detection and Surveillance (IDDS), and Association of Public Health Laboratories (APHL) of the United States, as well as local community organizations (21).

## Responding to the Second Wave of the Pandemic: the SARS-COV-2 Laboratory Diagnosis Network

The need to expand laboratory testing capacity in 2020 was responded to by further increasing the number of referral laboratories to 685 (22); which included animal health laboratories, drug and food laboratories, and mobile PCR. In parallel, the samples tested increased. Specifically, by November 2020, the number of referral laboratories had increased about 10 times as compared to April 2020, with the number of specimens tested increased by 65 times, and the number of people tested increased by 56 times. However, the laboratory accrual rate remained vulnerable to external pressures, such as a shortage of reagents and equipment; lack of qualified human resources; importation and procurement times.

One year since the first case was reported in Indonesia, there were 685 laboratories across 34 provinces (as of end-February 2021). This list included laboratories equipped only with PCR machines (558), TCM machines (70), and having both PCR and TCM machines (57). The top five provinces with the highest number of laboratories were West Java (104), DKI Jakarta (97), East Java (90), Central Java (49), and North Sumatera (23), reflecting the provinces with largest populations. These laboratories comprise of laboratories that belong to different ministries and institutions, including the Ministry of Health; Ministry of Education, Culture and Higher Education; Subnational Governments; Indonesian Private Hospitals Association; Ministry of Research and Technology; Food and Drug Supervisory Agency; Ministry of Agriculture; Ministry of State Owned Enterprises; the State Police of the Republic of Indonesia; the Indonesian National Armed Forces; Ministry of Religion and the Indonesian Civil Servant Corps Foundation (Supplementary Information). This highlights the cross-ministerial collaboration as a critical factor in strengthening and expanding the laboratory network, since these unprecedented demands were well-beyond the capacities of any single organization. Additionally, this allowed for services complementarity and for an equitable distribution of referral laboratories across 34 provinces.

## Beyond the Second Wave: COVID-19 Testing Progress in Indonesia

**Figure 1:**
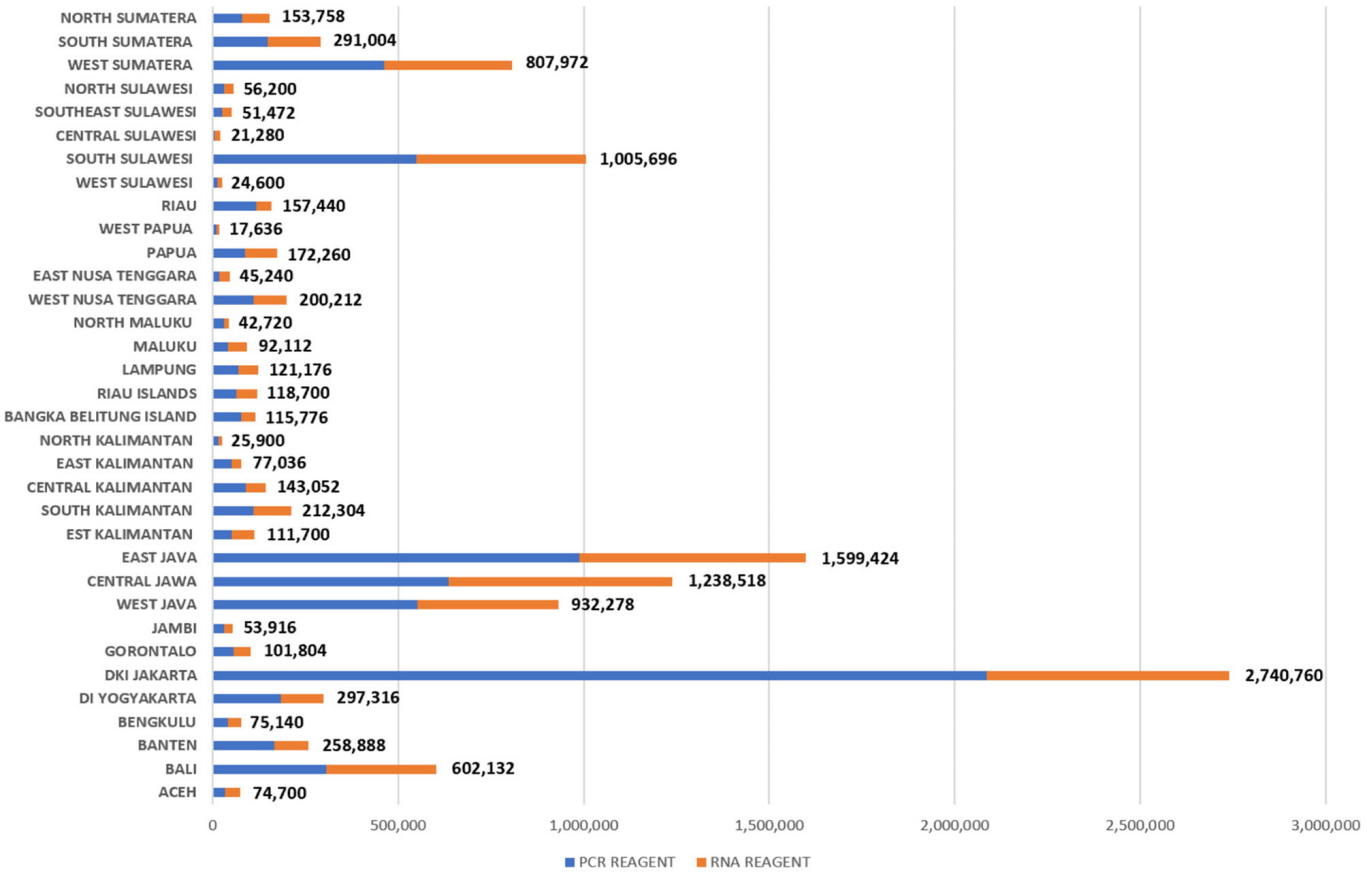
Distribution of PCR and RNA reagents in Indonesia as per March 6th, 2021 (21).

Figures 1A,B show the numbers of tests, and people tested, respectively, over time in Indonesia. By the end of February 2021, the monthly average of specimens tested was 56,015 and of people tested was 37,519.

The WHO standard recommends that 1,000 people per one million population per week are tested. This requires Indonesia to test around 270 thousand people per week from its ~270 million population. It was adopted as the national indicator for it offers a globally useful benchmark, despite the slow process to achieve it due to the constrained resources. Specifically, on the first week of June 2020, Indonesia only met 16.86% of this target, in August 2020 it reached 46.46%, it was significantly increased to 70% in September 2020 and to 88.66% in November. This target was finally achieved at 100% in mid-January 2021, and has been steadily maintained afterwards (Figure 1).

Province-wise, West Java has the highest number of people that need to be tested every week (45,161 people), followed by East Java (40,479) and Central Java (36,364). Detailed data on testing needs per province are provided on Supplementary Table 1. Supplementary Table 1 also shows that there was a significant variance of each province's ability to respond to the needs, against a background of uneven population distribution and a complex decentralized national-subnational governance, where district and city governments within each province can adopt their own contextual policies that differ from the national government.

**Figure 2:**
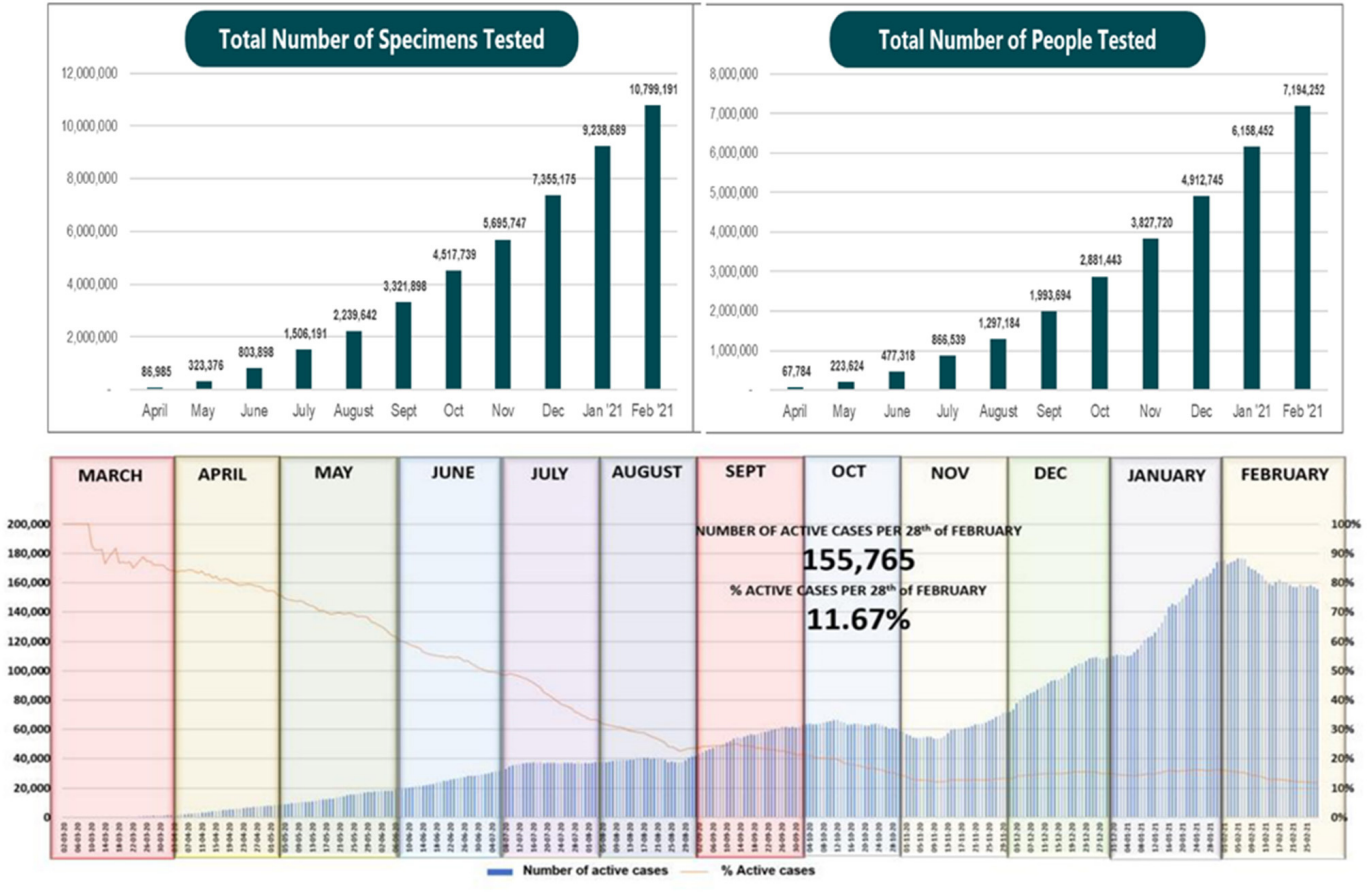
(**A**, Upper Left) total number of specimens tested in Indonesia June 2020 - February 2021; (**B**, Upper Right) Total number of people tested in Indonesia June 2020-February 2021 (24); (Below) the number of active cases reported in Indonesia per month (March 2020-February 2021).

Another limiting factor was the logistics support; particularly the distribution of reagents whose different levels of demand per province are indicated on Figure 2. This exposed Indonesia's persisting logistic transportation issues due to the highly geographically fragmented archipelago that spans across a distance equivalent to that from London to Moscow (25). In response, the procurement of reagents was allowed to be done by sub-national governments, with additional assistance where needed by the national government. By the end of February 2021, the National COVID-19 Handling Task Force distributed 92 PCR machines; 7,323,512 sets of PCR reagents; 4,697,439 Viral Transport Medium vials; and 9,650 PCR Kits for GeneXpert [the latest test for tuberculosis (TB)] across Indonesia (26).

## Challenges and Opportunities from the COVID-19 Laboratory Diagnostics Experiences

The most persistent challenge during the first 12 months of the bandemic was the unequal distribution of appropriately trained human resources across the 34 provinces. Even when the logistics support chain was addressed and equipment/reagents were made available, the lack of trained staff hindered laboratories from reaching their maximum outputs. This was also highlighted in previous pandemics (27–29), and while it was addressed to some extent, it was not sufficient for the unprecedented scale of the current pandemic. Fortunately, Indonesia's peaking demographic with an increasing share of a population in the “productive age” (15–59 years old) provided an opportunity to supply the numbers of workforce needed in many sectors (30), including health, though it leaves a question on the existing educational training and workforce linkage.

Secondly, the pressures from the pandemic provided an opportunity to reflect on and assess existing surveillance systems, where additional diagnostic capacity was required; and how existing infrastructure was utilized by other infectious disease surveillance programs, such as TB and Hepatitis. This was one of the key aspects allowing the successful increase of diagnostic capacity, as previous surveillance efforts had laid the foundation for additional infectious disease surveillance capabilities nationally in a harmonized manner. However, this a coordination problem which may stay for the longer-term. It is important to consider that COVID-19 testing should not (out) compete routine diagnostics when, for example, laboratory budgets and resources need to be reallocated over a wider operational period (31).

Thirdly, geographical and administrative fragmentation has been well-known to affect emergency logistical distribution, including reagents in Asia during SARS outbreak in 2003 (23) and Southeast Asia during H5N1 outbreak in the 2000s (32). Following previous natural and public health emergencies, Indonesia tried addressing a number of aspects to reduce the potential impact of this fragmentation during national emergencies (33, 34). Facing a similar issue with Indonesia, Malaysia learned from previous emergencies to build trust among different government and non-governmental institutions for strong cross-sectoral collaborations to ease logistics distribution pressures during the COVID-19 pandemic (35).

Fourthly, the involvement of laboratories from many different governmental departments and the private sector under one reporting structure demonstrates a successful effort to integrate what was siloed before. However, there are further improvements that can be made, for example while the reporting for COVID-19 testing is in real-time, the consumables requests are not. This can potentially limit smooth operations over the longer-term. Additionally, overlapping laboratory logistical records may affect the speed of distribution, as local governments can procure logistics themselves, or ask the Task Force or the MoH directly or both. In the latter case it would be difficult to validate competing requests because such data are not integrated.

Finally, the variety of laboratory equipment in Indonesia presents another challenge in terms of the laboratory response. Many equipment belonged to siloed projects, initiatives and/or collaborations with international organizations. Operating those under a harmonized protocol might not be possible if more complex laboratory tests are needed. They also present a logistics challenge, because reagents are not necessarily interchangeable across different equipment. The auditing process by the national government was able to harmonize operations across different platforms. Perhaps the pandemic would be a timely opportunity to review and address this aspect in a more systematic way.

## Conclusion

This ongoing pandemic has tested the preparedness, response and resilience of the laboratory systems in Indonesia for over 1 year. Indonesia is one of the countries where diagnostic laboratory readiness is still not sufficient, particularly with an uneven distribution of staff, equipment, and consumables capacities across a number of provinces. Thus, coping with the existing pandemic presented a real challenge, especially in terms of a consistent increase in testing capacity. This is common in resource restricted settings, especially in countries with large population and geographical fragmentation.

The Indonesian government made concerted efforts to foster collaboration across sectors, thus increasing testing capacity and reaching 685 designated laboratories within the first 12 months of the pandemic. However, existing laboratory facilities, reporting structures and the availability of qualified human resources remain concerning in the effort to increase testing capacity with a uniform quality. These challenges exposed some public health issues left to be addressed, which can in turn provide opportunities for future infectious diseases surveillance in Indonesia and countries in similar settings.

## Author Contributions

All authors listed have made a substantial, direct and intellectual contribution to the work, and approved it for publication.

## Conflict of Interest

GI and LM are affiliated with Aceso Global Health Consultants Ltd, which is a private company, we declare that this research project does not receive funding from Aceso Global Health Consultants. The company does not have a role in the study design, data collection and analysis, decision to publish, or preparation of the manuscript. LM is the director of the company, and GI is an employee of the company. However, both of them contributed to this paper on a pro bono basis. The remaining authors declare that the research was conducted in the absence of any commercial or financial relationships that could be construed as a potential conflict of interest.

## Abbreviations

APHL: Association of Public Health Laboratories of the United States
CDC: United States Centers for Disease Control and Prevention
COVID-19: Coronavirus Disease 2019
DKI &#8211 Jakarta: Daerah Khusus Ibukota - Special Region of the Capital City Jakarta
IDDS: Infectious Disease Detection and Surveillance
MERS: Middle East Respiratory Syndrome
MoH: Ministry of Health &#8211 of the Republic of Indonesia
NIHRD: National Institute of Health Research and Development
PCR: Polymerase Chain Reaction
SARS-CoV-2: Severe Acute Respiratory Syndrome Corona Virus 2
TCM: Tes Cepat Molekuler - Molecular Rapid Test (TCM)
TB: Tuberculosis
UNICEF: United Nations Children's Fund
USAID: United States Agency for International Development
WHO: World Health Organization.

## References

[1] Buño Soto A. The role of laboratory medicine specialists in the COVID-19 pandemic. Adv Lab Med Av En Med Lab. (2020) 1:1–2. 10.1515/almed-2020-0037 PMC10158743 37363781

[2] Pizzol JLD Hora VP da Reis AJ Vianna J Ramis I Groll A von. Laboratory diagnosis for Covid-19: a mini-review. Rev Soc Bras Med Trop. (2020) 53:e20200451. 10.1590/0037-8682-0451-2020 32876316 PMC7451498

[3] Dramé M Tabue Teguo M Proye E Hequet F Hentzien M Kanagaratnam L. Should RT-PCR be considered a gold standard in the diagnosis of COVID-19? J Med Virol. (2020) 92:2312–3. 10.1002/jmv.25996 32383182 PMC7267274

[4] Russo A Minichini C Starace M Astorri R Calò F Coppola N. Current status of laboratory diagnosis for COVID-19: a narrative review. Infect Drug Resist. (2020) 13:2657–65. 10.2147/IDR.S264020 32801804 PMC7413717

[5] Smith DW. The challenges of establishing adequate capacity for SARS-CoV-2 testing. Med J Aust. (2020) 212:457–8. 10.5694/mja2.50610 32401351 PMC7272790

[6] Algaissi AA Alharbi NK Hassanain M Hashem AM. Preparedness and response to COVID-19 in Saudi Arabia: Building on MERS experience. J Infect Public Health. (2020) 13:834–8. 10.1016/j.jiph.2020.04.016 32451260 PMC7211706

[7] Kim J-H An JA-R Oh SJ Oh J Lee J-K. Emerging COVID-19 Success Story: South Korea Learned the Lessons of MERS. Exemplars in Global Health. Available online at: https://www.exemplars.health/emerging-topics/epidemic-preparedness-and-response/covid-19/south-korea (accessed June 28, 2021).

[8] Durant TJS Peaper DR Ferguson D Schulz WL. Impact of COVID-19 pandemic on laboratory utilization. J Appl Lab Med. (2020) 5:1194–205. 10.1093/jalm/jfaa121 32663258 PMC7454564

[9] Wu C Chen X Cai Y Xia J Zhou X Xu S. Risk factors associated with acute respiratory distress syndrome and death in patients with coronavirus disease 2019. Pneumonia in Wuhan, China. JAMA Intern Med. (2020) 180:934–43. 10.1001/jamainternmed.2020.0994 32167524 PMC7070509

[10] Zhou F Yu T Du R Fan G Liu Y Liu Z. Clinical course and risk factors for mortality of adult inpatients with COVID-19 in Wuhan, China: a retrospective cohort study. Lancet. (2020) 395:1054–62. 10.1016/S0140-6736(20)30566-3 32171076 PMC7270627

[11] Li C Zhao C Bao J Tang B Wang Y Gu B. Laboratory diagnosis of coronavirus disease-2019 (COVID-19). Clin Chim Acta. (2020) 510:35–46. 10.1016/j.cca.2020.06.045 32621814 PMC7329657

[12] Burki T. Understanding variants of SARS-CoV-2. Lancet. (2021) 397:462. 10.1016/S0140-6736(21)00298-1 33549181 PMC7906644

[13] COVID-19 Dashboard. Johns Hopkins Center for System Science and Engineering (JHU CSSE). (2021). Available online at: https://gisanddata.maps.arcgis.com/apps/opsdashboard/index.html#/bda7594740fd40299423467b48e9ecf6 (accessed June 28, 2021).

[14] European Centre for Disease Prevention and Control. COVID-10 Situation Update Worldwide, as of Week 8. (2021). Available online at: https://www.ecdc.europa.eu/en/geographical-distribution-2019-ncov-cases (accessed June 28, 2021).

[15] Aisyah DN Mayadewi CA Diva H Kozlakidis Z Siswanto Adisasmito W. A spatial-temporal description of the SARS-CoV-2 infections in Indonesia during the first six months of outbreak. PLoS ONE. (2020) 15:e0243703. 10.1371/journal.pone.0243703 33351801 PMC7755207

[16] Komisi Penanganan COVID-19 dan Pemulihan Ekonomi Nasional. Peta Sebaran COVID-19. (2021). Available online at: https://covid19.go.id/peta-sebaran-covid19 (accessed June 28, 2021).

[17] Axil Scientific. RNA Extraction for COVID-19. Available online at: https://axilscientific.com/fight-covid-19-with-axil/rna-extraction (accessed June 28, 2021).

[18] Hendarwan H Syachroni S Aryastami N Su'udi A Susilawati M Despitasari M. Assessing the COVID-19 diagnostic laboratory capacity in Indonesia in the early phase of the pandemic. WHO South East Asia J Public Health. (2020) 9:134–40. 10.4103/2224-3151.294307 32978346

[19] Ministry of Health Republic of Indonesia. Keputusan Menteri Kesehatan KMK No.HK.01.07/Menkes/182/2020 Tentang Jejaring Laboratorium Pemeriksaan COVID-19. Jakarta (2020).

[20] Ministry of Health Republic of Indonesia. Keputusan Kementerian Kesehatan Republik Indonesia Nomor HK.01.07-MENKES-405-2020 tentang Jejaring Laboratorium Pemeriksa. COVID-19. Jakarta (2020).

[21] Ministry of Health Republic of Indonesia. Intra Action Review COVID-19: Indonesia. Jakarta (2020).

[22] National Institute of Health Research and Department Ministry of Health Republic of Indonesia. Daftar Laboratorium Pemeriksa Covid-19. Available online at: https://www.litbang.kemkes.go.id/laboratorium-pemeriksa-covid-19/ (accessed June 28, 2021).

[23] Lau Y Jiamian Z Ng Adolf KY Panahi R. Implications of a pandemic outbreak risk: a discussion on China's emergency logistics in the era of coronavirus disease (2019). (COVID-19). J Int Logist Trade. (2020) 18:127–35. 10.24006/jilt.2020.18.3.127

[24] Bidang Data dan IT Satuan Tugas Penanganan COVID-19. Analisis Data COVID-19 Indonesia (Update Per 28 Februari 2021). (2021). Available online at: https://covid19.go.id/p/berita/analisis-data-covid-19-indonesia-update-28-februari-2021 (accessed June 28, 2021).

[25] Arnquist S Weintraub R. HIV/AIDS in Indonesia: Building a Coordinated National Response. Boston, MA: Harvard Business Publishing (2011).

[26] Badan Nasional Penanggulangan Bencana (BNPB -Indonesian National Agency for Disaster Countermeasures). Indonesian Situation Report. (2021). Available online at: https://web.bnpb.go.id/pusdalops/pages/data (accessed July 3, 2021)

[27] Zumla A Goodfellow I Kasolo F Ntoumi F Buchy P Bates M. Zika virus outbreak and the case for building effective and sustainable rapid diagnostics laboratory capacity globally. Int J Infect Dis. (2016) 45:92–4. 10.1016/j.ijid.2016.02.1007 26952389 PMC7129671

[28] Curley M Thomas N. Human security and public health in Southeast Asia: the SARS outbreak. Aust J Int Aff. (2004) 58:17–32. 10.1080/1035771032000184737

[29] Wertheim HFL Puthavathana P Nghiem NM van Doorn HR Nguyen TV Pham HV. Laboratory capacity building in Asia for infectious disease research: experiences from the South East Asia Infectious Disease Clinical Research Network (SEAICRN). PLoS Med. (2010) 7:e1000231. 10.1371/journal.pmed.1000231 20386725 PMC2850380

[30] Oey-Gardiner M Gardiner P. Indonesia's demographic dividend or window of opportunity. Masy Indones. (2013) 39:481–504. Available online at: http://jmi.ipsk.lipi.go.id/index.php/jmiipsk/article/viewFile/626/419 (accessed June 28, 2021).

[31] Vandenberg O Martiny D Rochas O van Belkum A Kozlakidis Z. Considerations for diagnostic COVID-19 tests. Nat Rev Microbiol. (2021) 19:171–83. 10.1038/s41579-020-00461-z 33057203 PMC7556561

[32] Ferguson NM Cummings DAT Cauchemez S Fraser C Riley S Meeyai A. Strategies for containing an emerging influenza pandemic in Southeast Asia. Nature. (2005) 437:209–14. 10.1038/nature04017 16079797

[33] Joyce N. Civilian-military coordination in the emergency response in Indonesia. Mil Med. (2006) 171:66–70. 10.7205/MILMED.171.1S.66 17447628

[34] Bisri MBF. Examining inter-organizational network during emergency response of West Java Earthquake 2009. Indonesia. Procedia Environ Sci. (2013) 17:889–98. 10.1016/j.proenv.2013.02.107

[35] Ab Malik MH Omar EN Maon SN. Humanitarian Logistics: a disaster relief operations framework during pandemic Covid-19 in achieving healthy communities. Adv Bus Res Int J. (2020) 6:101–13. 10.24191/abrij.v6i2.11114

