# Supplementary material for: Laboratory Readiness and Response for SARS-Cov-2 in Indonesia
Source: Front Public Health. 2021 Jul 19;9:705031. doi: 10.3389/fpubh.2021.705031 (PMC8326463; doi:10.3389/fpubh.2021.705031)
Supplement: Supplementary file 1 [file Table_1.DOCX]

**Supplementary Table S1**. Number of people tested per one million of population across 34 provinces in Indonesia

| **Provinces** | **WHO benchmark** | **Number of People Tested per 1 Million Population** | | | | |
| --- | --- | --- | --- | --- | --- | --- |
|  |  | **31-Oct** | **07-Nov** | **14-Nov** | **21-Nov** | **28-Nov** |
| Aceh | 5,247 | 251 | 214 | 231 | 379 | 345 |
| Bali | 4,216 | 1,264 | 966 | 1,027 | 1,603 | 1,466 |
| Bangka Belitung Isles | 1,379 | 884 | 534 | 594 | 919 | 683 |
| Banten | 10,722 | 1,144 | 981 | 1,518 | 1,539 | 970 |
| Bengkulu | 1,999 | 360 | 138 | 169 | 443 | 365 |
| Central Java | 36,364 | 860 | 625 | 809 | 1,416 | 1,329 |
| Central Kalimantan | 2,570 | 1,059 | 679 | 696 | 1,116 | 876 |
| Central Sulawesi | 2,955 | 255 | 53 | 116 | 116 | 222 |
| DI Yogyakarta | 3,631 | 732 | 937 | 1,247 | 1,054 | 1,631 |
| DKI Jakarta | 10,846 | 5,989 | 5,075 | 5,624 | 5,867 | 7,151 |
| East Kalimantan | 3,522 | 3,098 | 2,426 | 2,912 | 3,653 | 3,584 |
| East Nusa Tenggara | 5,411 | 173 | 92 | 138 | 145 | 285 |
| Gorontalo | 1,180 | 764 | 387 | 456 | 1,100 | 443 |
| Jambi | 3,493 | 428 | 195 | 339 | 467 | 445 |
| West Java | 45,161 | 668 | 489 | 668 | 789 | 798 |
| East Java | 40,479 | 926 | 640 | 715 | 820 | 854 |
| Lampung | 9,095 | 162 | 169 | 216 | 244 | 376 |
| Maluku | 1,847 | 548 | 70 | 486 | 379 | 791 |
| North Kalimantan | 648 | 522 | 723 | 312 | 768 | 584 |
| North Maluku | 1,307 | 827 | 642 | 420 | 877 | 990 |
| North Sulawesi | 2,641 | 1,003 | 635 | 785 | 1,513 | 1,673 |
| North Sumatera | 14,874 | 596 | 612 | 483 | 604 | 688 |
| Papua | 4,340 | 1,070 | 1,020 | 1,408 | 2,004 | 1,745 |
| Riau | 6,074 | 838 | 922 | 939 | 1,294 | 2,144 |
| Riau Islands | 1,929 | 625 | 405 | 645 | 846 | 623 |
| South Kalimantan | 4,023 | 1,359 | 671 | 899 | 1,419 | 1,152 |
| South Sulawesi | 9,426 | 354 | 475 | 639 | 1,042 | 771 |
| South Sumatera | 8,217 | 447 | 361 | 437 | 335 | 620 |
| Southeast Sulawesi | 2,635 | 284 | 145 | 426 | 414 | 331 |
| West Kalimantan | 5,422 | 493 | 583 | 376 | 827 | 861 |
| West Nusa Tenggara | 5,270 | 232 | 119 | 115 | 358 | 238 |
| West Papua | 1,140 | 282 | 720 | 1,078 | 1,398 | 2,142 |
| West Sulawesi | 1,559 | 50 | 73 | 861 | 1,234 | 316 |
| West Sumatera | 5,519 | 1,007 | 1,769 | 767 | 1,736 | 1,989 |

Remarks:

Xxxxxxx : has achieved WHO’s standard
