# Supplementary figures and images for: Laboratory Readiness and Response for SARS-Cov-2 in Indonesia
Source: Front Public Health. 2021 Jul 19;9:705031. doi: 10.3389/fpubh.2021.705031 (PMC8326463; doi:10.3389/fpubh.2021.705031)

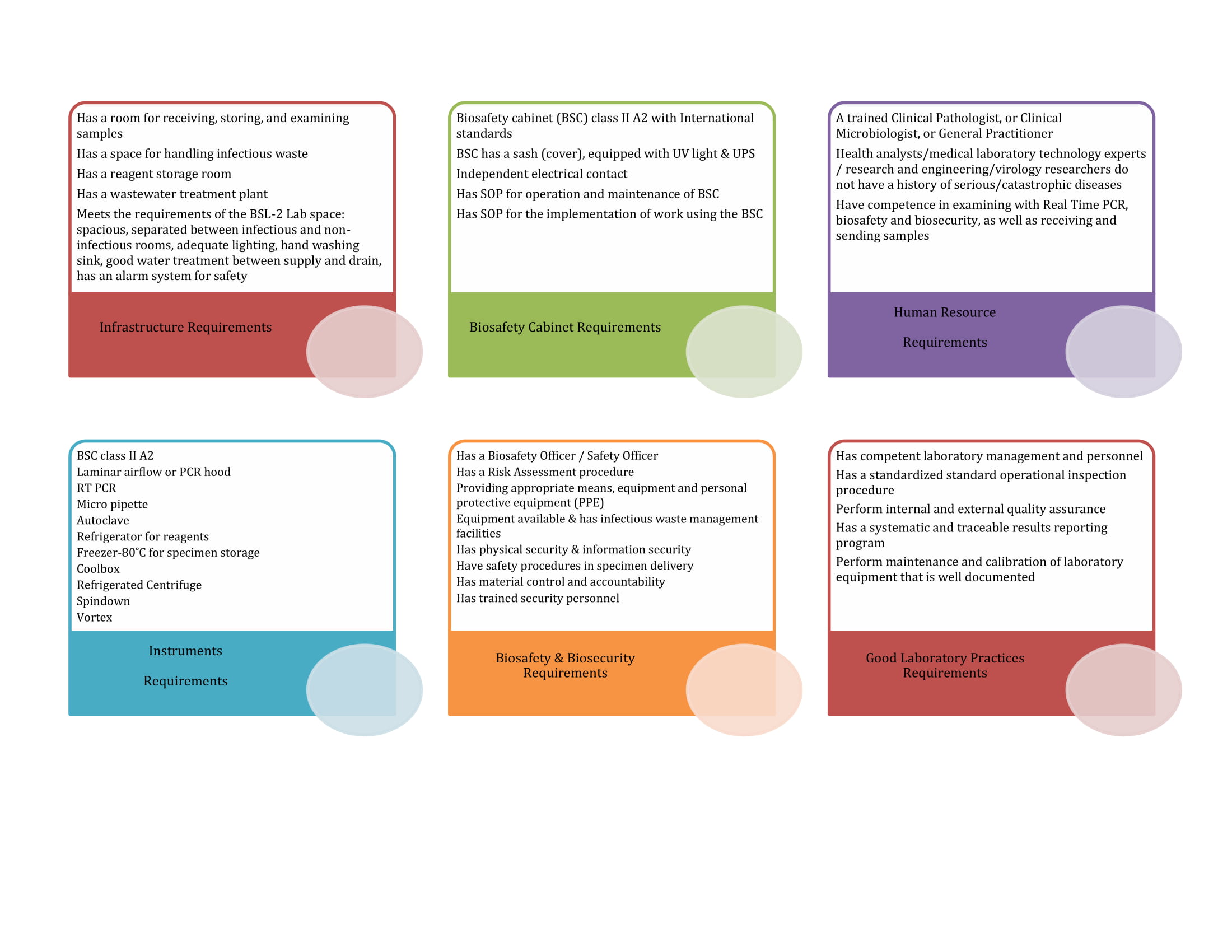

Supplement: Supplementary file 2 [file Image_1.JPEG]

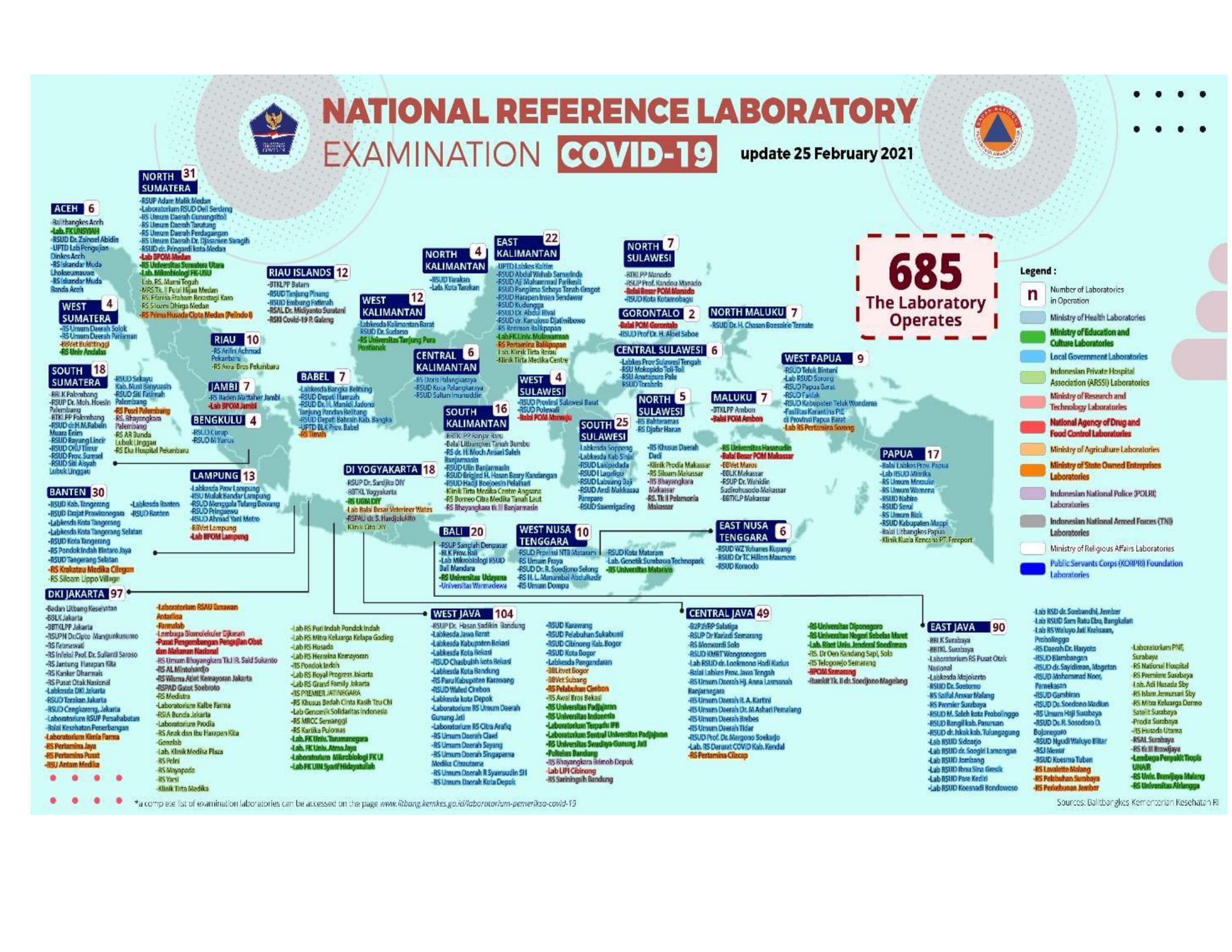

Supplement: Supplementary file 3 [file Image_2.JPEG]

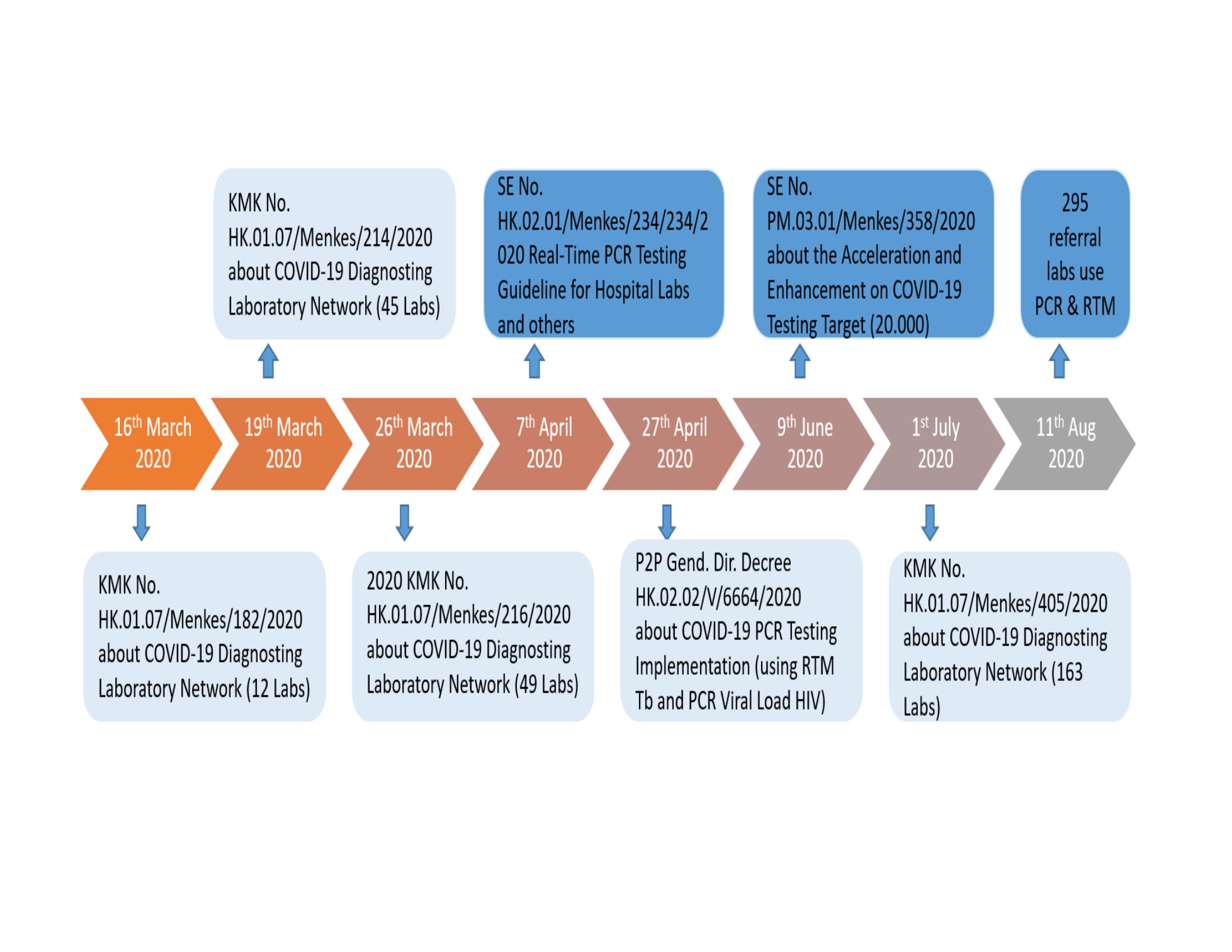

Supplement: Supplementary file 4 [file Image_3.JPEG]

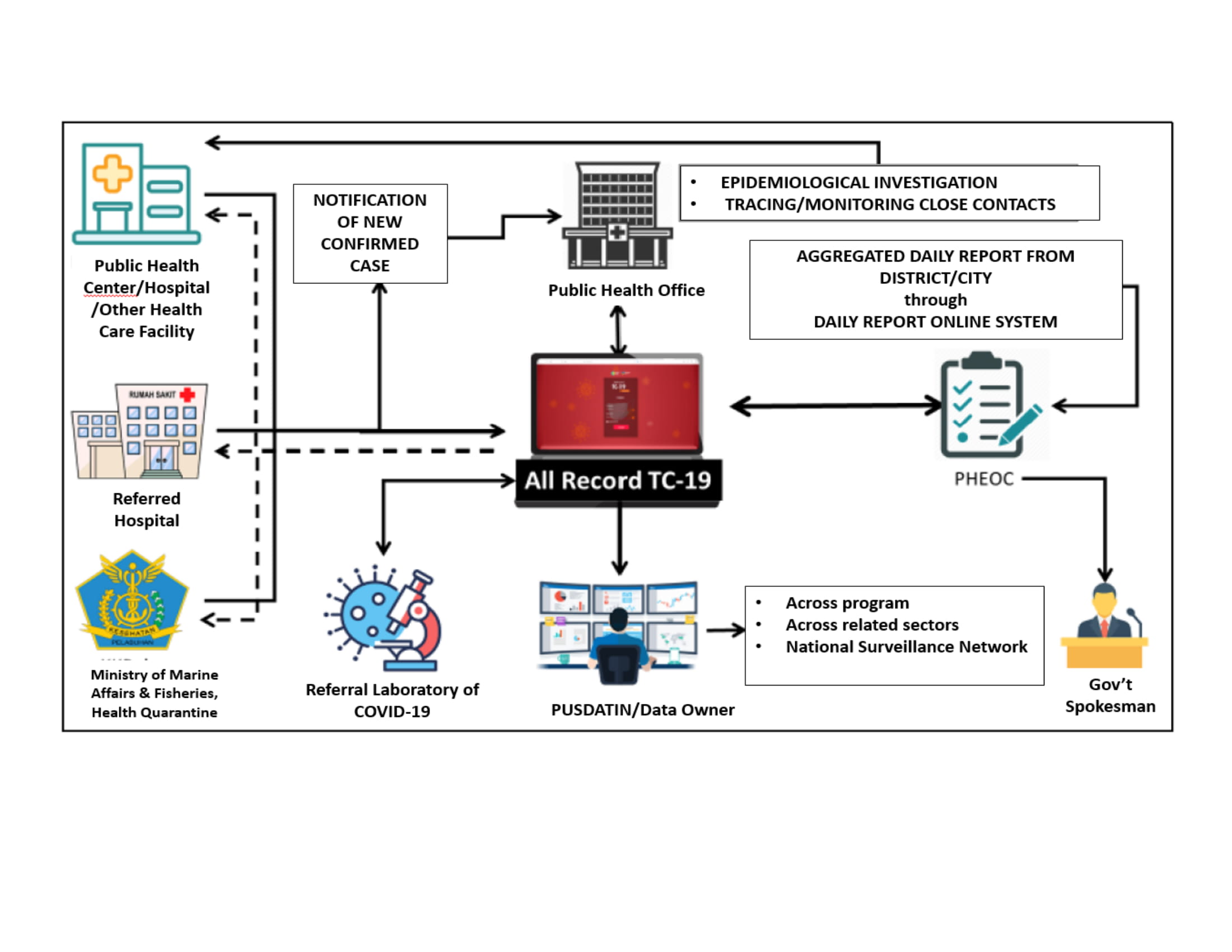

Supplement: Supplementary file 5 [file Image_4.JPEG]
